# Targeting CAM-DR and Mitochondrial Transfer for the Treatment of Multiple Myeloma

**DOI:** 10.3390/curroncol29110672

**Published:** 2022-11-09

**Authors:** Rikio Suzuki, Daisuke Ogiya, Yoshiaki Ogawa, Hiroshi Kawada, Kiyoshi Ando

**Affiliations:** Department of Hematology/Oncology, Tokai University School of Medicine, Isehara 259-1193, Japan

**Keywords:** multiple myeloma, MM niche, CAM-DR, tunneling nanotube, mitochondrial transfer

## Abstract

The prognosis of patients with multiple myeloma (MM) has improved dramatically with the introduction of new therapeutic drugs, but the disease eventually becomes drug-resistant, following an intractable and incurable course. A myeloma niche (MM niche) develops in the bone marrow microenvironment and plays an important role in the drug resistance mechanism of MM. In particular, adhesion between MM cells and bone marrow stromal cells mediated by adhesion molecules induces cell adhesion-mediated drug resistance (CAM-DR). Analyses of the role of mitochondria in cancer cells, including MM cells, has revealed that the mechanism leading to drug resistance involves exchange of mitochondria between cells (mitochondrial transfer) via tunneling nanotubes (TNTs) within the MM niche. Here, we describe the discovery of these drug resistance mechanisms and the identification of promising therapeutic agents primarily targeting CAM-DR, mitochondrial transfer, and TNTs.

## 1. Introduction

Multiple myeloma (MM) is a B-cell hematologic malignancy characterized by abnormal proliferation of plasma cells in the bone marrow microenvironment (BMM), monoclonal protein (M protein), hypercalcemia, renal dysfunction, anemia, and lytic bone lesions [1,2]. MM accounts for approximately 10% of newly diagnosed hematological malignancies [3]. With the advent of innovative analytical technologies in the 2000s, research to elucidate the molecular pathology of MM has intensified [4]. This has led to the rapid development and clinical introduction of many novel molecular-targeted therapies, including proteasome inhibitors (PI), immunomodulatory drugs, and immunotherapies, which have dramatically improved the prognosis of patients with MM [5]. However, relapse is highly likely in almost all MM patients; thus, there is an urgent need to develop next-generation therapeutic agents that could cure MM [6].

MM develops a myeloma niche (MM niche) in the BMM. Data clearly indicate that MM cells modify the microenvironment to facilitate their survival. Research also indicates that the BMM plays an important role in the drug resistance mechanism of MM. In particular, adhesion between MM cells and BM stromal cells mediated by adhesion molecules induces cell adhesion-mediated drug resistance (CAM-DR) [7]. Analyses of the role of mitochondria in cancer cells, including MM cells, has revealed that the mechanism leading to drug resistance involves the exchange of mitochondria between cells (mitochondrial transfer) via tunneling nanotubes (TNTs) within the MM niche [8,9,10,11]. Elucidation of these mechanisms has facilitated the development of many therapeutic agents targeting CAM-DR, mitochondrial transfer, and TNTs, and it is expected that more will be developed in the future.

The purpose of this review is to summarize research findings regarding the MM niche, CAM-DR, and TNTs and discuss the treatment methods targeting these mechanisms that are currently under development or have been clinically applied.

## 2. Hematopoietic Stem Cell (HSC) Niche and MM Niche

Various pathways and cell types have been shown to tightly control the self-renewal, proliferation, and differentiation properties of HSCs in normal hematopoiesis and development of the HSC niche [12,13,14]. In particular, the HSC niche consists of a cellular component (hematopoietic and nonhematopoietic or stromal cells, such as osteoblasts, osteoclasts, fibroblasts, adipocytes, myocytes, endothelial cells, lymphocytes, dendritic cells, and macrophages), extracellular matrix (several types of collagen, laminin, fibronectin, thrombospondin, proteoglycans, and hemonectin), and a soluble component (cytokines, growth factors, and soluble isoforms of cell adhesion molecules (e.g., serum vascular cell adhesion protein 1, serum intercellular adhesion molecule 1, sP-selectin, and sE-selectin)), and it undergoes appropriate remodeling by osteoclasts [15,16,17,18,19]. Importantly, cancer cells, including MM cells, have been shown to engraft in the endosteal HSC niche, invade bone, and induce tumor expansion and metastatic disease [12,16,20,21,22]. Especially in terms of MM, research clearly shows that osteoclasts, vascular endothelial cells, and BM stromal cells play important roles in creating the suitable environment for MM cells referred to as the MM niche [23]. In the MM niche, MM cells alter the normal HSC niche and induce the expression of specific cytokines and growth factors that promote their survival, growth, and drug resistance [12].

Interactions between MM cells and the MM niche, either directly through cell adhesion molecule-mediated interactions between MM cells and bone marrow stromal cells (BMSCs), or indirectly via the effects of growth factors released by both cell types, miRNA, or mitochondrial transfer, activate a pleiotropic proliferative and antiapoptotic cascade [24]. Importantly, the adhesion of MM cells to BMSCs and/or the extracellular matrix triggers the NF-κB-dependent transcription and secretion of cytokines such as IL-6, tumor necrosis factor-α, and osteopontin in BMSCs, which further stimulates development of drug resistance or so-called cell adhesion-mediated drug resistance (CAM-DR) [24,25,26]. Notably, CAM-DR plays a significant role in the development of drug resistance in MM [7,26,27]. Therefore, targeting CAM-DR is now thought to be a promising option to improve the prognosis of MM patients. 

## 3. CAM-DR Components as Druggable Targets

**Figure 1:**
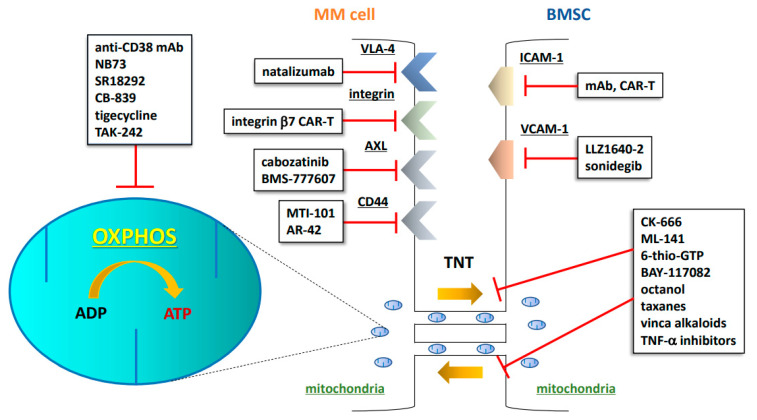
Schematic illustration of CAM-DR, OXPHOS, and TNTs as druggable targets in the MM niche. MM: multiple myeloma; BMSC: bone marrow stromal cell; mAb: monoclonal antibody; CAR-T: chimeric antigen receptor T cell; TNT: tunneling nanotube; OXPHOS: oxidative phosphorylation; ADP: adenosine diphosphate; ATP: adenosine triphosphate; VLA-4: very late antigen-4; AXL: AXL receptor tyrosine kinase; ICAM-1: intercellular adhesion molecule-1; VCAM-1: vascular cell adhesion molecule-1; TNF-α: tumor necrosis factor-α.

The development of targeted therapies for CAM-DR is an area of growing interest [26]. CAM-DR is induced by adhesion molecules such as integrin family members [28,29], CD138 (syndecan-1) [28], CD44 [28], vascular cell adhesion molecule-1 (VCAM-1) [30], lymphocyte function-associated antigen-1 (LFA-1) [31,32], and intercellular adhesion molecule-1 (ICAM-1) [33]. Therefore, considerable research is currently focused on the development of drugs targeting these molecules (Figure 1 and Table 1) [34].

Integrins play crucial roles in adhesion, migration, invasion, BM homing, survival, proliferation, and drug resistance in MM cells [37,59]. In particular, very late antigen-4 (VLA-4) (α4β1) and α4β7 play pivotal roles in the pathophysiology of MM [59], thus making these molecules attractive targets. Considering this background, natalizumab, a recombinant humanized IgG4 monoclonal antibody that binds integrin-a4, has demonstrated an ability to inhibit the adhesion of MM cells to both noncellular and cellular components of the MM niche [35,36]. Notably, Hosen et al. reported that integrin β7 is constitutively activated in MM cells, and chimeric antigen receptor (CAR) T cells targeting integrin β7 exhibit a superior anti-MM effect [37,38].

CD44 is a ubiquitous surface molecule, as well as a member of the glycoprotein family [60]. Importantly, CD44 variants are highly expressed in MM cells derived from extramedullary lesions, which play a role in the mechanism underlying the refractoriness of MM [61]. MTI-101, a first-in-class peptidomimetic, binds CD44/ITGA4-containing complexes to induce the activation of Stim1 and TRPC1 expression, triggering necrotic cell death of MM cell lines. MTI-101 and related peptidomimetics are, thus, regarded as an attractive class of compounds [40,41]. Considerable research has focused on eliciting anti-MM effects by modulating CD44 expression. Canella et al. reported that the pan-histone deacetylase inhibitor AR-42 downregulated CD44 expression and enhanced the anti-MM activity of lenalidomide in primary MM cells isolated from lenalidomide-resistant patients and cells isolated from an in vivo MM mouse model [42].

VCAM-1 is an endothelial ligand for VLA-4 (or α4β1) of the β1 subfamily of integrins, and it has been implicated as playing a role in the homing and migration of MM cells [62,63,64]. Teramachi et al. reported that inhibition of TGF-β-activated kinase-1 using LLZ1640-2 reduces VCAM-1 expression in BMSCs and impairs MM cell adhesion to BMSCs [45]. In addition, Zhang et al. reported that the Hedgehog inhibitor LDE225 (sonidegib) inhibits MM cell proliferation by blocking Hedgehog signaling and modulates stromal cells within the BMM by decreasing the expression of VCAM-1 and other adhesion molecules, suggesting that Hedgehog inhibition is a promising option for the treatment of MM [46,47].

LFA-1 is an adhesion molecule that mediates lymphocyte adhesion [65]. The LFA-1 inhibitor LFA878 exerts an anti-MM effect via inhibition of the LFA-1/FAK/PI3K/AKT axis [65]. Importantly, LFA-1 is gaining increased attention for its potential to modulate the tumor microenvironment (TME). The inability of CD8+ effector T cells in the TME is an important mechanism of immunotherapy resistance [66]. Hickman et al. elegantly demonstrated that activation of LFA-1 mediated by the small-molecule LFA-1 activator 7HP349 converts a T-cell-exclusionary TME to a T-cell-enriched TME [66]. Therefore, this activator could be a promising candidate drug for the treatment of MM.

The overexpression of ICAM-1 in MM, associated with advanced disease and poor survival, may be a potential therapeutic target even in the relapse/refractory setting [33,67,68,69]. Sherbenou et al. reported that an anti-ICAM-1 monoclonal antibody conjugated to an auristatin derivative induced potent anti-MM cytotoxicity both in vitro and in vivo. This effect was assumed to involve in part blockade of cell–cell interactions and the interaction of ICAM-1 with its ligand, thus interfering with various immune functions [33]. Furthermore, a line of anti-ICAM-1 antibody-based chimeric antigen receptor T cells was shown to exhibit significant antitumor effects both in vitro and in vivo in preclinical models of gastric cancer and thyroid cancer, suggesting they are applicable to the treatment of hematological malignancies, including MM [33,43,44].

The HSC niche can regulate the dormancy of tumor cells [21]. In MM, dormancy occurs when tumor cells enter a quiescent state (G0), in which they are under reversible growth arrest [70,71]. Importantly, dormant MM cells can be induced to re-enter the cell cycle in response to extrinsic stimuli from the microenvironment or various therapeutic agents, including bortezomib [71,72]. Drug-resistant dormant MM cells residing in skeletal endosteal niches are thought to mediate disease relapse. These cells exhibit a distinct transcriptome signature enriched in immunity-related genes and genes associated with myeloid cell differentiation, including AXL (a TAM receptor tyrosine kinase). Notably, AXL inhibition using the small-molecule inhibitors cabozatinib and BMS-777607 releases MM cells from dormancy and sensitizes them to chemotherapy [16,39]. Another study found that macrophages are the dominant cells regulating the inflammatory milieu of the MM niche; inhibition of TPL2 kinase in macrophages leads to inhibition of interleukin (IL)-1β and IL-6, ultimately resulting in myeloma progression [73]. These data could lead to the development of new therapies that improve of the outcome of MM patients.

## 4. Mitochondrial Transfer via TNTs: A Novel CAM-DR Concept

### 4.1. Mitochondrial Transfer in Cancer Cells, including MM Cells

It is now clear that the metabolic and mitochondrial functions are reprogrammed in many types of cancer cells to ensure the production of necessary molecules such as lipids, proteins, and nucleic acids and sustain the mitotic signaling that enables cell proliferation [74]. Mitochondria generate most of a cell’s energy supply, i.e., adenosine triphosphate (ATP), via oxidative phosphorylation (OXPHOS) [8]. Cancer cells tend to synthesize ATP primarily through glycolysis, even under aerobic conditions, although glycolysis is less efficient than OXPHOS in generating ATP [75]. However, cells of certain solid tumors and many hematological malignancies appear to exhibit normal or even increased OXPHOS and mitochondrial metabolism [76]. However, this remains an area of intense research, as the association between cancer cells and OXPHOS has not been fully elucidated.

Studies have clearly demonstrated the occurrence of mitochondrial transfer in hematological malignancies such as acute myeloid leukemia, acute lymphocytic leukemia, and MM [8,9,10,11,77]. Transcellular mitochondrial transfer is primarily mediated by three intercellular communication pathways: (1) TNTs, (2) extracellular vesicles, and (3) gap junctions [76]. Interestingly, cancer cells can transfer mitochondria to nonmalignant cells via mitophagy, which is a process for the clearance of damaged mitochondria [78]. Significantly, the transfer of mitochondria and/or mitochondrial DNA to cancer cells increases the mitochondrial content and enhances OXPHOS, thus favoring proliferation and invasion [8]. The transfer of mitochondria from BMSC was shown to protect mutant hematopoietic cells during chemotherapy [8]. Thus, mitochondrial exchange occurs preferentially between nonmalignant cells and cancer cells. Cancer cells that acquire mitochondria exhibit chemoresistance [79], suggesting that this process is a promising target in the treatment of various cancers, including MM.

### 4.2. TNT Formation in Cancer Cells, including MM Cells

TNTs are filamentous, F-actin-rich, long tubular extensions connecting the cytoplasm of adjacent and/or distant cells that mediate cell-to-cell communication [80,81,82]. TNTs are increasingly considered the primary intercellular pathway for the unidirectional and bidirectional movement of nuclear and cytoplasmic cargo, such as nucleic acids, drugs, pathogenic molecules, and organelles, including mitochondria [50,83]. Hypoxic conditions associated with the TME reportedly stimulate an increase in TNT formation [84]. Under conditions of oxidative stress, the intracellular expression of p53 is upregulated, and protein kinase B–phosphoinositide 3-kinase–mammalian target of rapamycin (AKT–PI3K–mTOR) signaling is activated, leading to TNT formation [76]. The mechanism of TNT formation is closely associated with interactions between a complex of proteins, including leukocyte specific transcript 1, M-sec, Ras-related protein A, and the exocyst complex [50,85,86,87]. TNT can rescue diseased cells and tissues by mediating the direct transfer of healthy mitochondria to compromised cells [88]. Mitochondrial transfer occurs via TNTs and partial cell fusion, and the process is significantly upregulated in the presence of chemotherapeutic drugs [89]. Importantly, transferred mitochondria were found to metabolically promote OXPHOS [11,52]. MM cells can acquire mitochondria from neighboring nonmalignant cells through TNTs. Moreover, TNT-mediated transfer from cancer cells also plays a role in drug resistance, as demonstrated by the detoxifying removal of chemotherapeutic agent-loaded lysosomal vesicles from leukemia cells [90,91]. Therefore, targeting mitochondrial transfer via TNTs is an attractive option for overcoming chemo-resistance in the treatment of cancers, including MM.

### 4.3. OXPHOS and TNTs as Druggable Targets in Cancer and MM Therapy

Numerous compounds have been identified that affect pathways, such as NF-κB and mTOR, or block actin polymerization, thus inducing a reduction in TNT formation. The compounds include cytochalasin D, cytarabine, latrunculin A and B, daunorubicin, everolimus, metformin, nocodazole CK-666, ML-141, 6-thio-GTP, BAY-117082, and octanol (Figure 1) [48,49]. In addition, taxanes and vinca alkaloids have the potential to partially inhibit mitochondrial transfer by inhibiting microtubule polymerization [50]. M-sec, a TNT marker and regulator of TNT formation, directly induces tumor necrosis factor (TNF)-α. These data suggest that TNF-α inhibitors could be used to indirectly reduce TNT formation, thereby inhibiting mitochondrial transfer [50,51].

Targeting mitochondrial respiration and OXPHOS is also an attractive treatment option (Figure 1). FOXM1 regulates the metabolism of myeloma cells by upregulating glycolysis and OXPHOS. NB73, a small-compound inhibitor of FOXM1, inhibits MM cell growth by promoting FOXM1 degradation, suggesting that NB73 could become a promising OXPHOS-targeted drug [53]. Xiang et al. reported that the expression of OXPHOS-associated genes is associated with higher PGC-1α expression; treatment with the PGC-1a inhibitor SR18292 was shown to significantly impair the proliferation and survival of MM cells due to dysfunction in OXPHOS metabolism [54]. Thompson et al. reported that PI-resistant MM cells exhibit an increased capacity for and reliance on mitochondrial respiration [55]. The glutaminase-1 inhibitor CB-839 inhibits mitochondrial respiration and is more cytotoxic to PI-resistant cells, suggesting that mitochondrial respiration would be a promising target in the treatment of relapsed/refractory MM [55]. The OXPHOS inhibitor tigecycline increases the sensitivity of cancer cells to bortezomib, a representative PI [56,57]. Inhibition of PGC-1a by SR18292 was shown to significantly impair the proliferation and survival of MM cells due to energy exhaustion and oxidative damage [54,56]. Toll-like receptor 4 (TLR4) induces mitochondrial biogenesis and an increase in mitochondrial mass in human MM cells. Moreover, bortezomib (BTZ) exposure activates TLR4 signaling in BTZ-resistant MM cell lines. Combining BTZ with the selective TLR4 inhibitor TAK-242 was shown to overcome drug resistance by inducing more intense and extended oxidative stress, leading to mitochondrial depolarization and severe impairment of mitochondrial fitness [58].

In the MM niche, TNT-mediated transcellular transfer of mitochondria from neighboring BMSCs to MM cells supports OXPHOS in MM cells, and this process is dependent on CD38 expression [76]. CD38 is a transmembrane glycoprotein present both on the cell membrane and in the intracellular compartment [92]. MM cells express high levels of CD38. Therefore, monoclonal antibodies against CD38 (e.g., datatumumab and isatuximab) can be used to successfully treat MM [92]. Anti-CD38 monoclonal antibodies have several mechanisms of action, including antibody-dependent cellular cytotoxicity, antibody-dependent cellular phagocytosis, complement-dependent cytotoxicity, direct cellular apoptosis, and modulation of extracellular ectoenzyme activity [93]. Importantly, MM patients receiving anti-CD38 antibody therapy have shown superior survival benefit. Nevertheless, MM cells may eventually acquire resistance to anti-CD38 antibody therapy in these patients [94]. Increased CD38 expression facilitating mitochondrial transfer from BMSCs to primary MM cells is one potential resistance mechanism [50,52]. CD38 expression blockade was shown to inhibit mitochondrial transfer, reduce tumor volume, and increase overall survival in mice [50,52]. These reports suggest that TNT inhibition using anti-CD38 antibodies may be a useful anti-myeloma therapy.

## 5. Conclusions

With the advent of novel therapeutic drugs, especially monoclonal antibody immunotherapies and CAR-T therapies, the prognosis of patients with MM has improved dramatically. However, MM cells typically eventually develop resistance; therefore, elucidation of the mechanisms via which these cells acquire resistance is urgently needed.

Research has shown that MM cells acquire drug resistance via contact with BMM constituents such as BMSCs. In particular, CAM-DR plays an important role in this process, and therapeutic agents that overcome resistance mediated by CAM-DR are, thus, being developed. Furthermore, recent data indicate that drug resistance is dynamically induced through mitochondrial transfer between MM cells and other BMM cells via TNTs, providing additional new therapeutic targets. Successful application of these therapies in clinical practice could bring us one step closer to making MM a curable disease.

## Figures and Tables

**Table 1:** Summary of drugs/treatments targeting CAM-DR, OXPHOS, and TNTs.

Druggable Targets	Drugs/Treatments	Description	References
VLA-4	natalizumab	Recombinant humanized IgG4 monoclonal antibody that binds integrin-a4.	[35,36]
integrin	integrin-b7 CAR-T	CAR-T cells targeting activated integrin-β7.	[37,38]
AXL	cabozatinib	Small-molecule multiple tyrosine kinases inhibitor.	[16,39]
	BMS-777607	Small-molecule c-Met/AXL inhibitor.	[16,39]
CD44	MTI-101	First-in-class peptidomimetic that binds CD44/ITGA4-containing complexes.	[40,41]
	AR-42	Pan-histone deacetylase inhibitor that downregulates CD44 expression.	[42]
ICAM-1	mAb	Anti–ICAM-1 mAb conjugated to an auristatin derivative.	[33]
	CAR-T	Anti–ICAM-1 antibody–based CAR-T cells.	[33,43,44]
VCAM-1	LLZ1640-2	TGF-β–activated kinase-1 inhibitor that reduces VCAM-1 expression.	[45]
	sonidegib	Hedgehog inhibitor that blocks Hedgehog signaling and decreases the expression of VCAM-1 and other adhesion molecules.	[46,47]
TNT	CK-666	Actin polemerization inhibitor that inhibits TNT formation.	[48,49]
	ML-141	Cdc42 GTPase inhibitor that decreasess TNT formation.	[48,49]
	6-thio-GTP	Vac-1-Rac signaling inhibitor that decreases TNT formation.	[48,49]
	BAY-117082	IkB/IKK inhibitor that decreasess TNT formation.	[48,49]
	octanol	Prevents TNT-mediated cell-cell communication.	[48,49]
	taxanes	Partially inhibits mitochondrial transfer by inhibiting microtubule polymerization.	[50]
	vinca alkaloids	Partially inhibits mitochondrial transfer by inhibiting microtubule polymerization.	[50]
	TNF-a inhibitors	Indirectly reduces TNT formation and thereby inhibit mitochondrial transfer.	[50,51]
OXPHOS	anti-CD38 mAb	Inhibits mitochondrial transfer and OXPHOS.	[50,52]
	NB73	Small-compound inhibitor of FOXM1 that promotes FOXM1 degradation and downregulates OXPHOS.	[53]
	SR18292	PGC-1a inhibitor that induces dysfunction in OXPHOS metabolism.	[54]
	CB-839	Glutaminase-1 inhibitor that inhibits mitochondrial respiration.	[55]
	tigecycline	Glycycline antibiotic that inhibits OXPHOS.	[56,57]
	TAK-242	TLR4 inhibitor that induces more intense and extended oxidative stress, leading to mitochondrial depolarization and severe impairment of mitochondrial fitness.	[58]
